# Impact of critical care environment on burnout, perceived quality of care and safety attitude of the nursing team**^1^**

**DOI:** 10.1590/1518-8345.1472.2884

**Published:** 2017-06-05

**Authors:** Edinêis de Brito Guirardello

**Affiliations:** 2 PhD, Associate Professor, Faculdade de Enfermagem, Universidade Estadual de Campinas, Campinas, SP, Brazil.

**Keywords:** Patient Safety, Quality of Health Care, Health Facility Environment, Nursing, Critical Care, Job Satisfaction

## Abstract

**Objective:**

assess the perception of the nursing team about the environment of practice in critical care services and its relation with the safety attitude, perceived quality of care and burnout level.

**Method:**

cross-sectional study involving 114 nursing professionals from the intensive care unit of a teaching hospital. The following instruments were used: Nursing Work Index-Revised, Maslach Burnout Inventory and the Safety Attitude Questionnaire.

**Results:**

the professionals who perceived greater autonomy, good relationships with the medical team and better control over the work environment presented lower levels of burnout, assessed the quality of care as good and reported a positive perception on the safety attitude for the domain job satisfaction.

**Conclusion:**

the findings evidenced that environments favorable to these professionals' practice result in lower levels of burnout, a better perceived quality of care and attitudes favorable to patient safety.

## Introduction

In the past three decades, considerable knowledge has been produced on the relevance of the environment of nursing practice for the nurse, patient and institution. The healthcare environment is complex, demands technological and human resources to respond to the patients and families' care demands and the paramount activity of nursing, representing the main contingent of health professionals, is patient surveillance 24 hours per day^1^. 

The environment of practice is defined as the organizational characteristics of a work environment that make the nursing practice easier or more difficult^2^. The American Association of Critical Care Nurses acknowledges the inseparable link between the quality of the work environment, the excellence of nursing practice and the patient and family care outcomes, highlighting six main standards to establish and sustain a healthy work environment, as follows: communication skills, cooperation among team members, effective decision making by the nurses, appropriate number of professionals, acknowledgement of the nurse for his/her contribution and authentic leadership by nurse leaders^3^.

The Institute of Medicine in the United States identified many risks to patient safety, originating in each component and level of the care system, including: work process, burden and work hours and work environment of the nursing team. It was equally highlighted that the changes in the care provision system in the past decades have affected the way nursing delivers care and preserves the patient safety^1^.

In this context, various studies focus on the impact of the environment of practice and the results for the patient and nursing, showing that environments in which the nurse possesses autonomy, control over the environment and good relationships with the medical team result in lower levels of burnout^4^ ^-^ ^6^, greater job satisfaction^5^ ^,^ ^7^, lesser intent to abandon the job^5^ ^,^ ^8^ and better outcomes for the patients in terms of care quality^5^ ^,^ ^9^ and patient safety^5^ ^,^ ^8^ ^,^ ^10^.

It is highlighted that, in Brazil, research on this theme is recent. Evidences appoint that environments favorable to nursing practice entail lower levels of emotional exhaustion for the professionals, greater job satisfaction and lesser intent to abandon the job^11^ ^-^ ^12^. Nevertheless, no studies are available that assess the impact of the environment of practice on the burnout level, job satisfaction and safety attitude of the nursing team at critical care services.

The objective in this study was to assess the perceptions of the nursing team about the environment of practice in critical care services and its relation with the safety attitude, perceived quality of care and burnout level.

## Method

A cross-sectional study was undertaken at three intensive care units (ICU) of a teaching hospital in the city of Campinas, State of São Paulo. The three ICUs are located in separate physical facilities, the first with 22 beds, divided in three nursing stations: neurology (seven beds), coronary unit (five beds) and general ICU (ten beds). The second, the Clinical Emergency and Trauma ICU, offers 20 beds distributed among three nursing stations, being ten beds for clinical emergency and ten for trauma victims. The third ICU, with eight beds, is the Transplantation ICU.

The nursing team at these units consists of nurses, nursing technicians, supervisors and a nurse manager. Except for the nurse manager, the nursing team members work thirty hours per week. For the study, all nurses and nursing technicians were eligible. The sample included the nursing professionals involved in direct patient care who had worked at the current place of work for three months or more. As exclusion criteria, the subjects were considered who were active in management activities or were absent from the unit due to holidays, leave of absence or sick leave.

Approval for the study was obtained from the institutional Research Ethics Committee (Opinion 512.574). The subjects who accepted to participate in the study signed the Informed Consent.

The researcher and an undergraduate nursing student grantee collected the data between December 2014 and February 2015, with the formal authorization of the institution's nursing director and approval from the institution's Research Ethics Committee. The professionals who complied with the inclusion criteria were individually contacted at the workplace. They received information on the research objective and those who agreed to participate in the study received an envelope with the Informed Consent and the data collection instruments. A date and time was set to return the instruments.

To collect the data, the following instruments were used: Nursing Work Index - Revised (NWI-R) - Brazilian version^13^ ^-^ ^14^, Brazilian version of the Safety Attitudes Questionnaire - Short Form 2006 (SAQ)^15^ ^)^ and the Maslach Burnout Inventory (MBI).

The Nursing Work Index - Revised (NWI-R), validated for the Brazilian culture, assesses the nursing professionals' perception of their work environment^11^. It consists of 15 items, distributed in four subscales: autonomy (five items), organizational support (ten items), control over the environment (seven items) and relations between physicians and nursing team (three items). It is highlighted that the items in the organizational support subscale were taken from the first three subscales and therefore are not added to the total number of items in the instrument. For the sake of this study, two versions of the NWI-R were considered, one for nurses^13^ and the other for nursing technicians^14^. Answers are provided on a four-point Likert scale, with the following alternatives: 1 (I strongly agree); 2 (I partially agree); 3 (I partially disagree) and 4 (I strongly disagree) and, the lower the score, the greater the presence of attributes favorable to these professionals' practice.

The Safety Attitudes Questionnaire Short Form 2006 - (SAQ) intends to assess the health professionals' perception of the safety attitudes permeating their work environment^15^. It contains 41 items, distributed in eight domains: job satisfaction, stress recognition, teamwork climate, safety climate, safe behavior, perception of unit management, working conditions and perception of hospital management.

Answers are provided on a five-point Likert scale, in which A = I strongly disagree, B = I somewhat disagree, C = neutral, D = I somewhat agree and E = I strongly agree. Another alternative answer is "X" = does not apply. The scores correspond to the average of the item scores in each subscale, with scores superior to 75 indicating the perception of a safe environment for the patient.

The objective of the Maslach Burnout Inventory (MBI) is to measure the professionals' physical and emotional exhaustion by assessing their feelings towards their own work. It contains 22 items, distributed in three subscales: emotional exhaustion (nine items), personal accomplishment (eight items) and depersonalization (five items). Answers are provided on a five-point Likert scale and the subjects are asked to answer how frequently they experience certain situations in their work environment, with the following alternative answers: 1 (never); 2 (rarely); 3 (sometimes); 4 (frequently) and 5 (always). The scores are obtained by adding up the item scores in each subscale, which are then ranked as low, moderate and high level based on the tertiles.

For the emotional exhaustion subscale, scores equal or inferior to 25 were considered low, superior to 25 and equal or inferior to 27 as moderate and superior to 27 as high level of burnout. For the depersonalization subscale, scores equal or inferior to 11 indicated low, superior to 11 and equal or inferior to 15 moderate, and superior to 15 high level of burnout. In the personal accomplishment subscale, scored inversely to the other subscales, scores equal or inferior to 33.5 were considered as high, superior to 33.5 and equal or inferior to 24 as moderate and superior to 33.5 as low level of burnout.

The characterization form contains the following variables: age, sex, length of professional experience, length of experience at the institution and current place of work. It also contains questions addressing the professionals' perception on the quality of care offered to the patient, on a scale ranging from very bad (1 point) to very good (4 points); the perceived adequacy of human resources, material and technological resources, with the alternatives yes and no and the professionals' intention to abandon nursing within the next 12 months was assessed by means of a visual analogue scale ranging from zero to ten in which, the higher the score, the greater the intent to abandon the profession.

The collected data were transferred to an electronic worksheet in Microsoft^(r)^ Office Excel 2007 and analyzed with the advice of a statistician, using the software SPSS^(r)^ 20.0 for Windows (Statistical Package for the Social Sciences version 20.0) and SAS 9.1.3 for Windows (Statistical Analysis System version 9.1.3).

Descriptive statistical analysis was applied to the subscales of the NWI-R, MBI, SAQ, personal and professional variables. Cronbach's alpha was used to assess the reliability of the instrument subscales. Coefficients can range between zero and one, with results equal or superior to 0.60 being considered satisfactory.

The correlations between the subscales of the NWI-R, MBI, SAQ and the other research variables were assessed by means of the Spearman correlation coefficient, which ranges between -1 and 1, analyzed according to the following criteria: absence of correlation (0.00), weak correlation (0.10 - 0.30), moderate correlation (0.31 - 0.50) and strong correlation (0.51 - 1.00). For the statistical tests, significance was set at 5%.

## Results

The study participants were 114 nursing professionals, 41 (36.0%) of whom were nurses and 73 (64.0%) nursing technicians. The professionals' average age was 35.4 years (Min=23; Max= 60, SD= 8.7 years), 75% of the sample being aged 41 years or younger. The majority was female (79.8%) and had only one job contract (81.4%).

What the professional education is concerned, 26 (63.4%) nurses held a specialization degree in intensive care or nursing management; 3 (2.63%) held a Master's degree; 1 (0.88%) a doctoral degree and 11 (26.8%) a Bachelor's degree. Among the nursing technicians, 27 (37.0%) held a Bachelor's degree in nursing.

These professionals' length of experience corresponded to 10.4 years (Min=2; Max= 32, SD= 6.2 years), length of experience at the unit 5.2 years (Min=0.5; Max= 26.3, SD=5.3 years) and length of experience at the institution 7.7 years (Min= 0.25 Max=30.3, SD= 7.4 years).

Most professionals assessed the quality of the care offered to the patients as good (77.2%) and very good (15.0%), was satisfied with the work (71.9%), agreed that the number of professionals who delivered care at the unit was appropriate (68.42%) and that the material and technological resources at the unit were appropriate in number and quality (59.65%). As regard the intention to abandon the profession, on a visual analogue scale from zero to ten, the average was 1.69 (Min=0.0; Max= 10, SD= 2.69). 

The nursing professionals' assessments of the environment of practice, burnout level and perceived safety attitude are displayed in Table 1. It is highlighted that, for the three measures, the reliability coefficients were satisfactory. For the NWI-R, the reliability coefficients corresponded to 0.70 for the autonomy subscale, 0.83 for relations between physician and nursing team and 0.73 for the control over the environment subscale. In the SAQ domains, the reliability coefficients ranged between 0.64 and 0.81. For the MBI subscales, these coefficients corresponded to 0.91 for the emotional exhaustion subscale, 0.79 for personal accomplishment and 0.73 for the depersonalization subscale. 

**Table 1:** Description of nursing professionals' perception for the subscales of the Nursing Work Index-Revised, Maslach Burnout Inventory and Safety Attitude Questionnaire. Campinas, SP, Brazil, 2014-2015.

Variables		Mean	SD	Minimum	First Quartile	Median	Third Quartile	Maximum
Nursing Work Index-Revised								
	Autonomy	1.93	0.53	1.00	1.60	1.80	2.20	3,60
	Organizational support	2.04	0.48	1.10	1.70	2.00	2.30	3,40
	Control over the environment	2.08	0.53	1.00	1.71	2.00	2.29	3,71
	Relations physicians and nursing team	2.10	0.65	1.00	1.67	2.00	2.33	4,00
Maslach Burnout Inventory								
	Emotional exhaustion	20.74	6.36	9.00	16.00	19.00	25.00	43,00
	Personal accomplishment	30.88	4.20	20.00	29.00	31.00	33.50	40,00
	Depersonalization	9.15	3.39	5.00	6.00	9.00	11.00	21,00
Safety Attitude Questionnaire								
	Job satisfaction	76.19	20.72	0.00	60.00	80.00	90.00	100,00
	Stress recognition	69.04	26.68	0.00	56.25	75.00	93.75	100,00
	Teamwork climate	68.62	17.22	20.83	54.17	70.83	79.17	100,00
	Safety climate	60.18	18.33	14.29	46.43	62.50	71.43	95,83
	Safe behavior	64.53	22.75	0.00	50.00	66.67	83.33	100,00
	Perception of unit management	56.53	22.51	0.00	50.00	58.33	70.83	100,00
	Working conditions	56.49	26.27	0.00	37.50	58.33	75.00	100,00

Next, we analyzed whether there were differences in the professionals' perception of the environment of practice, burnout, safety attitudes and perceived quality of care. It was verified that the professionals differed in their perceived safety attitude in relation to the stress recognition, one of the SAQ domains, in which the nurses scored higher than the nursing technicians (p = 0.0025).

In another analysis, we looked for correlations among the NWI-R subscales, SAQ domains, MBI subscales and the variable perceived quality of care (Table 2).

**Table 2:** Spearman correlation coefficient among subscales of the Nursing Work Index-Revised, subscales of the Maslach Burnout Inventory, subscales of the Safety Attitude Questionnaire and perceived quality of care (n= 114). Campinas, SP, Brazil, 2014-2015.

Variables	Autonomy	Relation physician nursing	Control over the environment	Emotional exhaustion	Personal accomplishment	Depersonalization	Teamwork climate	Safety climate	Job satisfaction	Stress recognition	Unit management	Hospital management	Working conditions	Safe behavior
Quality of care	-0.44*	-0.37*	-0.30**^†^**	-0.29**^†^**	0.33**^†^**	-0.22**^†^**	0.39*	0.39*	0.34**^†^**	-0.04	0.27**^†^**	0.19	0.20**^†^**	0.31**^†^**
Autonomy				0.41*	-0.25**^†^**	0.25**^†^**	-0.46*	-0.43*	-0.39*	0.15	-0.47*	-0.39*	-0.49*	-0.46*
Relation physician and Nursing				0.23**^†^**	-0.12	0.21**^†^**	-0.48*	-0.37*	-0.25**^†^**	0.15	-0.29**^†^**	-0.21**^†^**	-0.36**^†^**	-0.38*
Control over the environment				0.48*	-0.24**^†^**	0.18	-0.48*	-0.42*	-0.38*	0.15	-0.43*	-0.39*	-0.44*	-0.43*
Emotional exhaustion							-0.47*	-0.41*	-0.37*	0.18	-0.37*	-0.37*	-0.41*	-0.39*
Personal accomplishment							0.33**^†^**	0.31**^†^**	0.36*	-0.04	0.25**^†^**	0.27**^†^**	0.17	0.19**^†^**
Depersonalization							-0.29**^†^**	-0.20**^†^**	-0.30**^†^**	-0.08	-0.27**^†^**	-0.33**^†^**	-0.36**^†^**	-0.23**^†^**

*p < 0.0001

†p < 0.05

When evaluating the existence of correlations among the SAQ subscales, the length of experience at the unit and the intention to abandon the profession, a weak correlation was found between the length of experience at the unit and the safety climate (r= -0.24; p= < 0.0112), job satisfaction (r= -0.24; p= < 0.0108), perception of unit management (r= -0.26; p= < 0.0054) and perception of hospital management (r= -0.26; p= < 0.0067) domains.

## Discussion

In this study, the impact of the environment of professional practice, the burnout level, the perceived quality of care and the safety attitudes of nursing professionals at intensive care units was investigated.

The participants were young adult professionals, mostly female with a single job. Most nurses held some specialization degree, but few degrees were related with their activity area, appointing that no culture exists at the institution to encourage the professionals to seek qualification/specialization related to their own work. As regards the nursing technicians, 27 (37.0%) held an undergraduate degree in nursing, demonstrating the concern with or search for qualified education.

Most professionals assessed the quality of the care offered to the patients as good and very good. The positive assessment of the care quality converges with other findings in this study, in that the professionals were satisfied with their work and did not intend to abandon the profession, besides their positive perception on the adequacy of human, material and technological resources at the unit. Studies emphasize the important relation between perceived quality of care, job satisfaction and lesser intent to abandon the job^5^ ^,^ ^8^, which are influenced by the dimensions of the nurse's environment of practice.

In relation to the perception of the environment of practice, most professionals perceive their work environment positively for all NWI-R subscales, particularly autonomy, which obtained the highest average when compared to the other dimensions of the NWI-R. The assessment between the professional categories did not result in significant differences. These findings support a study at a critical care unit^16^, one of the few to assess the environment of practice at this type of unit.

What the burnout is concerned, the majority presented a low level of emotional exhaustion and feeling of depersonalization and a moderate level of personal accomplishment. In an earlier study, emotional exhaustion was an important variable, not only when assessed individually for the nurse, but also when measured between groups. As regards the unit, emotional exhaustion indicates the pressure nurses experience in the context of the team they work in and reflects the team's experience^17^. It is important to highlight that, in this research, the burnout level did not differ between the nurses and nursing technicians.

With regard to the perceived safety attitude, it could be identified that only the job satisfaction domain was considered favorable, as observed earlier^15^. The stress recognition and teamwork climate domains, on the other hand, obtained averages superior to 68 points and should be valued by the unit manager, as they can influence the perceived safety attitude at the institution.

Significant differences were verified between the professional categories in terms of stress recognition, as most nurses, corresponding to more than 3/4 of this group, presented averages of 93.75 points or higher, signaling that the perceived stress was an important variable that interferes in these professionals' perceived safety attitude.

As appointed in other international studies, a significant correlation was found between the NWI-R (autonomy, relations with physicians and control over the environment), emotional exhaustion and job satisfaction^4^ ^-^ ^5^ ^,^ ^7^ ^-^ ^8^ ^,^ ^17^.

The perceived quality of care resulted in a moderate and significant correlation with the subscales autonomy, relation between physician and nursing team, personal accomplishment and with the domains: teamwork climate, safety climate, job satisfaction and safe behavior. In other words, professionals with autonomy and good relationships with the medical team feel personally accomplished, report that they offer a good quality of care to the patient and that they have a positive safety attitude, as demonstrated by the teamwork climate, safety climate, job satisfaction and safe behavior.

Another important aspect similar to the international studies^8^ ^,^ ^18^ was the moderate correlation between emotional exhaustion and the subscales autonomy and control over the environment. This means that, in environments in which the professionals report having autonomy and control over the environment, they present lower levels of emotional exhaustion. In addition, a moderate and weak correlation was evidenced between the subscales: autonomy, control over the environment, good relationships between physician and nursing team and all SAQ domains, except for the stress recognition domain, which showed no correlation with any of the variables.

And finally, but not less important, a weak correlation was found between the length of experience at the unit and the safety climate, job satisfaction, perception of unit and hospital management domains, indicating that, the shorted the length of experience at the unit, the worse these professionals' perception of the safety attitude.

This is one of the first studies developed at intensive care units in Brazil to show that environments favorable to the professional practice, with perceived autonomy, good professional relationships and control over the environment result in job satisfaction and lower levels of burnout for the nursing professionals.

## Study limitations

This study comes with several limitations. The convenience sample does not permit the generalization of the data. The research scenario was a public teaching hospital and may not picture the reality of the intensive care units in the State of São Paulo and other Brazilian regions. Finally, intervention studies need to be developed to assess strategies that result in improvements in the environment of practice.

## Conclusion

The findings evidence that environments favorable to the nursing team's professional practice can result in lower levels of emotional exhaustion, a higher quality of care and a positive perception of the safety attitudes.
